# Genome-wide assessment of genetic diversity and population structure insights into admixture and introgression in Chinese indigenous cattle

**DOI:** 10.1186/s12863-018-0705-9

**Published:** 2018-12-20

**Authors:** Wengang Zhang, Xue Gao, Yang Zhang, Yumin Zhao, Jiabao Zhang, Yutang Jia, Bo Zhu, Lingyang Xu, Lupei Zhang, Huijiang Gao, Junya Li, Yan Chen

**Affiliations:** 1 Innovation Team of Cattle Genetics and Breeding, Institute of Animal Science (IAS), Chinese Academy of Agricultural Sciences (CAAS), Beijing, 100193 China; 2 Xinjiang Academy of Animal Science, Urumqi, 830011 China; 3 Jilin Academy of Animal Science, Changchun, 130124 China; 4 Jilin University, Changchun, 130012 China; 5 Institute of Animal Husbandry and Veterinary Medicine, AnhuiAcademyof Agricultural Sciences, Hefei, 230031 China

**Keywords:** Chinese indigenous cattle, Population structure, Genetic diversity

## Abstract

**Background:**

China exhibits a great diversity of ecosystems and abundant cattle resources, with nearly 30 million cattle from 53 indigenous breeds reared in specific geographic regions. To explore the genetic diversity and population structure of Chinese indigenous cattle, a population genetic analysis at both the individual and population levels was conducted and the admixture analysis was performed. We genotyped 572 samples from 20 Chinese indigenous cattle breeds using GeneSeek Genomic Profiler Bovine LD (*GGP-LD, 30 K*) and downloaded the published data of 77 samples from 4 worldwide commercial breeds genotyped with Illumina BovineSNP50 Beadchip (*SNP50, 50 K*).

**Results:**

In principal component analysis (PCA) and neighbour-joining (NJ) tree analysis, samples of the same breeds were grouped together, leading to clear separation from other breeds. And Chinese indigenous cattle were clustered into two groups of southern and northern breeds, originated from Asian *Bos indicus* lineage and Eurasian *Bos taurus* lineage, respectively. In STRUCTURE K = 2, a clear transition occurred from the northern breeds to the southern breeds. Additionally, the northern breeds contained a smaller Eurasian taurine (62.5%) descent proportion than that reported previously (more than 90%). In STRUCTURE K = 3, a distinct descent was detected in the southern Chinese breeds, which could reflect a long-term selection history of Chinese indigenous cattle. The results from TreeMix and *f3* statistic provided the evidence of an admixture history between southern breeds and northern breeds.

**Conclusions:**

Consistent with the observed geographical distributions, Chinese indigenous cattle were divided into two genetic clusters, northern indigenous cattle and southern indigenous cattle. Three improved breeds in the northern area also exhibited northern indigenous ancestry. We found that the breeds distributed in the northern China showed more southern lineage introgression than previously reported. Central-located populations appeared to the admixture between southern and northern lineages, and introgression events from European cattle were observed in Luxi Cattle, Qinchuan Cattle and Jinnan Cattle. The study revealed the population structures and levels of admixture pattern among Chinese indigenous cattle, shedding light on the origin and evolutionary history of these breeds.

**Electronic supplementary material:**

The online version of this article (10.1186/s12863-018-0705-9) contains supplementary material, which is available to authorized users.

## Background

Cattle is an important part of the agricultural economy worldwide, used mainly for milk, meat, and fur. Modern cattle are domesticated primarily from two primary areas, Eastern Europe and the Indian subcontinent [1, 2], resulting in two independent lineages, Eurasian taurine (or Eurasian *Bos taurus*) and Asian indicine (Asian *Bos indicus* or zebu). Controversially, another domestication event might have occurred to shape African taurine [3]. The domestication of cattle occurred as early as 8000 years ago [1, 4–6], leading to spatial dispersion due to human migration and ecological changes, and cattle subsequently underwent a recent rapid decrease in their effective population size in response to artificial selection and natural selection [4, 5, 7]. Long-term selection pressure has most likely operated on genomic regions resulting in a diversity of genetic backgrounds in worldwide cattle. Consequently, the formation of varieties brings a broad range of phenotypic variation (e.g., dairy cattle breeds [8] and polled beef cattle breeds [9]).

China has a great diversity of ecosystems and abundant cattle resources, including nearly 30 million indigenous cattle, which are reared in specific geographic regions. According to the Domestic Animal Diversity Information System (FAO-DAD-IS, http://www.fao.org/dad-is/), China has more than 70 cattle breeds, including 53 Chinese indigenous cattle breeds [10]. Indigenous breeds are divided into three primary groups on the basis of their geographic distributions and morphological characteristics: a northern-distributed group in north China, a central-distributed group in the middle and lower areas of the Yellow River, and a southern-distributed group in south China [11]. Studies on Y chromosome polymorphisms and mitochondrial DNA (mtDNA) sequences clearly demonstrated that Chinese indigenous cattle originated from both humpless breeds (Eurasian *Bos taurus*) and humped breeds (*Bos indicus*) [12–14] and revealed a declining south-to-north gradient of zebu introgression [15]. To specific, previous studies reported that cattle in northern China includes more than 90% *Bos taurus* component of total genome [15]. Analyses of ancient DNA indicated that domesticated cattle most likely first appeared in northern China between approximately 3000 BC and 2000 BC [16–19], or even longer ago [20], whereas cattle of the indicine lineage first appeared in the southern and central plains of China at least 1500 BC [21].

Originally raised for use as draft animals, Chinese indigenous cattle are generally employed for farm-related work or transportation, forming local environmental adaptation and physical endurance [10]. Southern cattle, which originated in mountainous areas, are generally resistant to damp, heat and mites and exhibit a small but robust and compact constitution. Northern cattle are generally cold and mite resistant, with thicker skins, coarser hair, sturdier bones, and broader chests than the southern breeds [10, 22, 23]. Additionally, the highland breed, Tibetan cattle, is adapted to the cold, oxygen-rarefied environments in high-elevation areas. Therefore, Chinese indigenous cattle represent genetic resources with specific traits such as powerful endurance, the ability to metabolize low-quality feed, and high disease resistance.

Genome-wide studies for estimating genetic diversity in cattle have been implemented using commercial SNP arrays [24–28]. In this study, we genotyped 572 Chinese cattle sampled among 20 breeds using the GeneSeek Genomic Profiler Bovine LD assay. We conducted principal component analysis (PCA), neighbour-joining tree (NJ tree) and STRUCTURE analyses to elucidate the population structure and genetic diversity, and performed TreeMix and 3 Population test (*f3*) analyses to explore the introgession events of Chinese indigenous breeds. Overall, this study helps to understand the genetic background and diversity of indigenous cattle and provides more detailed information on migration and introgression, shedding light on the origin and evolutionary history of Chinese breeds.

## Results

**Fig. 1:**
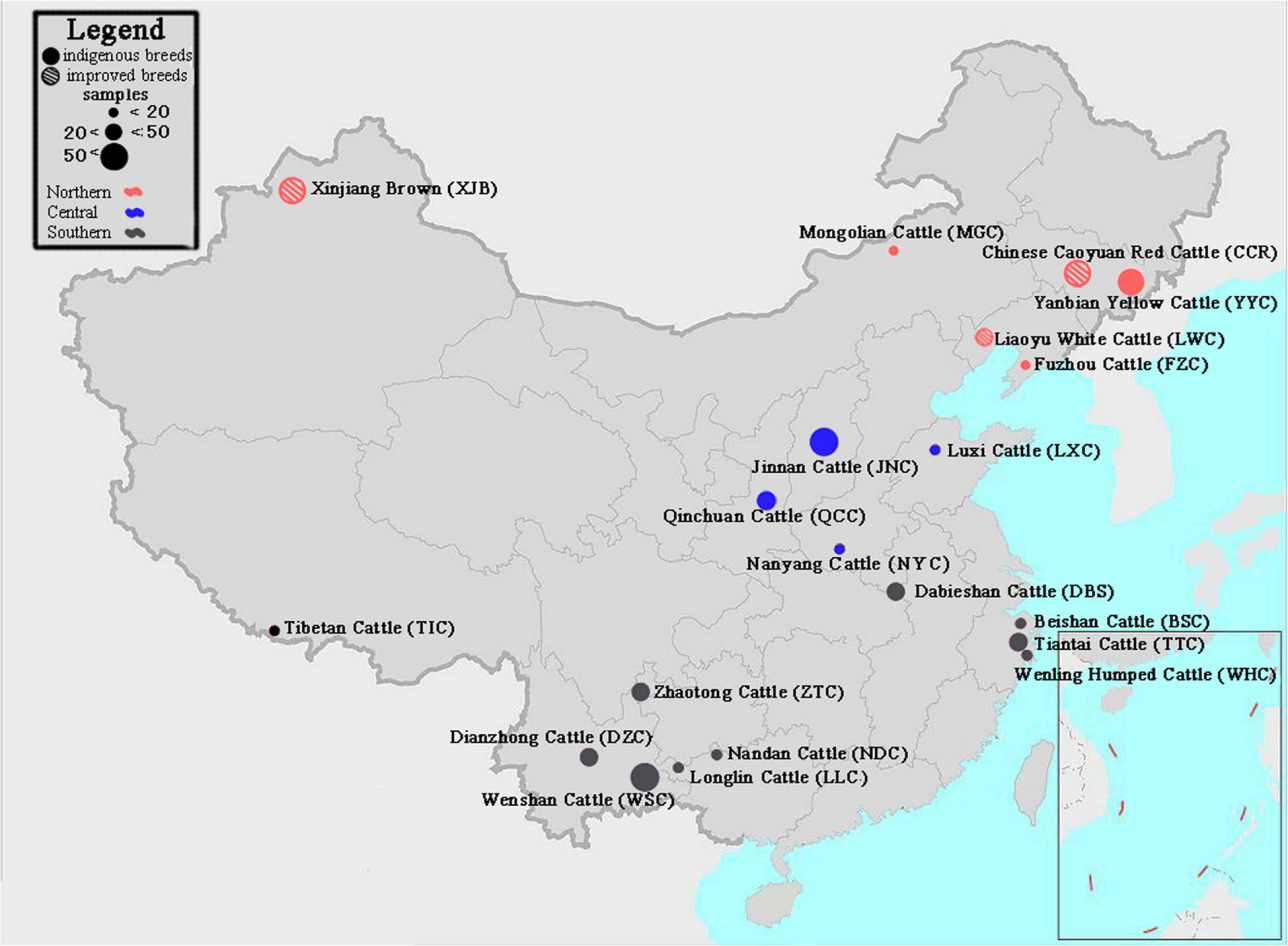
The distribution of indigenous cattle breeds on a map of China. A total of 20 Chinese cattle breeds were sampled in this study, including 17 indigenous cattle breeds and 3 improved breeds. Red, blue, and grey plots represent Northern-distribution, Central-distribution, and Southern-distribution breeds, respectively. (Map is downloaded from Wikimedia Commons https://commons.wikimedia.org/wiki/File:China_location_map.svg)

According to geographic dispersal, we divided all the samples into three groups (Fig. 1): northern-located breeds (Fuzhou (*n = 13*), Mongolian (*15*), Yanbian Yellow (*59*), Liaoyu White (*20*), Chinese Caoyuan Red (*26*), and Xinjiang Brown (*47*)); central-located breeds (Nanyang (*15*), Luxi (*14*), Qinchuan (*30*), and Jinnan (*55*)); and southern-located breeds (Beisha (*19*), Tiantai (*18*), Wenling Humped (*20*), Nandan (*19*), Longlin (*15*), Dianzhong (*30*), Wenshan (*47*), Zhaotong (*43*), and Dabieshan (*44*)). Additionally, only Tibetan (*20*) belonged to highland breeds. Detailed information on breed names, abbreviations, and distributions was listed in Table 1. Among the breeds, three improved breeds, LWC, CCR, and XJB, were the local cattle breed crossbred with European cattle and have undergone productive selection for a period of time, and other 17 breeds were Chinese indigenous cattle. All samples (*n = 572*) were genotyped with GeneSeek Genomic Profiler Bovine LD (*GGP-LD*) assays (*n = 30,125*). For interpretations in a global phylogeographic context, we also included previously published data from 77 individuals of four worldwide breeds [29] that were genotyped with the Illumina BovineSNP50 BeadChip (*SNP50*) [30]. Number of individuals for each breed were shown in Table 1. We merged common SNPs in *GGP-LD* and *SNP50*, obtained 7522 SNPs and then performed linkage disequilibrium SNP pruned (LD pruned) with 7003 SNPs for the following analysis. LD pruned was removal of one SNP from each pair where r^2^ > 0.1 within a 50-SNP window.

**Table 1:** Proportion of polymorphic SNPs, observed and expected heterozygosities, inbreeding coefficient and effective population size in Chinese and worldwide breeds

	Breed	Breed abbr.	*Nt* ^a^	*Nq* ^b^	Region^c^	Combined data set^i^					*Ne* ^j^
						*MAF* ^d^	*P*n^e^	*H*o^f^	*H*e^g^	*F* ^h^	
Chinese indigenous cattle breeds	Beisha	BSC	19	18	South	0.21	0.848	0.289	0.285	−0.015(0.015)	10
	Tiantai	TTC	21	21	South	0.21	0.859	0.280	0.284	0.016(0.031)	59
	Wenling Humped	WHC	20	19	South	0.22	0.893	0.300	0.297	−0.009(0.11)	72
	Nandan	NDC	19	19	South	0.21	0.834	0.275	0.279	0.013(0.078)	67
	Longlin	LLC	15	15	South	0.23	0.906	0.310	0.307	−0.011(0.406)	59
	Dianzhong	DZC	30	30	South	0.26	0.924	0.331	0.339	0.024(0.005)	29
	Wenshan	WSC	47	47	South	0.26	0.939	0.333	0.335	0.008(0.212)	152
	Zhaotong	ZTC	43	42	South	0.28	0.965	0.366	0.365	−0.004(0.511)	85
	Dabieshan	DBS	44	44	South	0.25	0.951	0.342	0.336	−0.019(0.001)	259
	Nanyang	NYC	15	13	Central	0.27	0.943	0.356	0.349	−0.021(0.02)	105
	Luxi	LXC	14	14	Central	0.28	0.962	0.361	0.368	−0.020(0.001)	221
	Qinchuan	QCC	30	30	Central	0.28	0.972	0.370	0.364	−0.018(0.001)	235
	Jinnan	JNC	55	55	Central	0.28	0.968	0.374	0.360	−0.038(0.001)	37
	Tibetan	TIC	20	7	Plateau	0.25	0.904	0.362	0.335	–	–
	Fuzhou	FZC	13	13	North	0.22	0.803	0.305	0.290	−0.051(0.05)	47
	Mongolian	MGC	15	15	North	0.25	0.904	0.331	0.322	−0.026(0.001)	206
	Yanbian Yellow	YYC	59	58	North	0.24	0.848	0.325	0.315	−0.032(0.001)	85
Improved cattle breeds	Chinese Caoyuan Red	CCR	26	26	North	0.25	0.849	0.331	0.316	−0.050(0.001)	63
	Liaoyu White	LWC	20	20	North	0.26	0.867	0.334	0.326	−0.023(0.001)	422
	Xinjiang Brown	XJB	47	47	North	0.22	0.769	0.313	0.288	−0.085(0.001)	29
Worldwide cattle breeds	Angus	AN	–	20	Scotland	0.30	0.971	0.395	0.386	−0.025(0.001)	
	Hereford	HFD	–	20	Wales	0.29	0.967	0.384	0.376	−0.022(0.001)	–
	GIR	GIR	–	20	India	0.16	0.737	0.227	0.225	−0.010(0.005)	–
	Sahiwal	SAHW	–	17	Pakistan	0.16	0.729	0.225	0.221	−0.019(0.012)	–
Mean						0.24	0.888	0.326	0.320	−0.019	118

^a^Total number of individuals sampled in each breed

^b^Number of individuals remaining after quality control, with a call rate > 0.95

^c^South refers to south China, Central refers to central China, North refers to north China and Plateau refers to Tibet

^d^Minor Allele Frequency

^e^Proportion of polymorphic loci within a breed

^f^Observed heterozygosity

^g^Expected heterozygosity

^h^Inbreeding coefficient (significant level)

^i^*P*n, *H*o, *H*e, and *F* are calculated based on 7003 SNPs using bootstrapping method

^j^Recent effective population size

### Genetic variation

Considering the influence of sample size and ascertainment bias of the beadchip design, we used a bootstrapping method to calculate the proportion of polymorphic SNPs (*P*n), observed heterozygosity (*H*o), expected heterozygosity (*H*e), inbreeding coefficient (*F*) and minor allele frequency (*MAF*) within each breed (Table 1).

In Chinese indigenous cattle, *P*n ranged from 0.769 (XJB) to 0.972 (QCC), with a mean of 0.895. One-way ANOVA analysis revealed that *P*n of central-located breeds (0.962) was significantly higher than that of northern-located breeds (0.840) (Additional file 1: Figure S1); however, no significant difference was observed between the southern-located breeds and central-located breeds or between the southern-located breeds and northern-located breeds. In terms of *MAF*, the average in southern, central and northern-located breeds was 0.24, 0.28, and 0.24, respectively, consistent with *P*n roughly (Additional file 1: Figure S2). As we all know, *H*o also reflects genetic diversity and history of a population. In this study, *H*o of NDC, TTC, and BSC were 0.275, 0.280, and 0.289, respectively, with relatively lower heterozygosity, whereas *H*o of JNC, QCC, and ZTC were 0.374, 0.370 and 0.366, respectively, with higher heterozygosity. For *F* values, 11 breeds (BSC, DBS, NYC, LXC, QCC, JNC, MGC, YYC, CCR, LWC, and XJB) had significantly negative values (*p* < 0.05), and only 2 breeds (TTC and DZC) were positive values (*p* < 0.05). The maximum value of *F* values was 0.024 in DZC, and the minimum was − 0.085 in XJB.

### Effective population size

In order to estimate contemporary effective population size (*N*e) for Chinese indigenous cattle using GGP-LD dataset, we used NeEstimator *V2*, a method based on linkage disequilibrium (LD). *N*e ranged from 10 to 422, with a mean of 118. BSC (10) exhibited the smallest estimated *N*e, suggesting a limited pool of BSC sires, whereas the improved breed LWC (422) presented the largest *N*e, suggesting much higher genetic diversity. *Ne* was not estimated for TIC because of the limitation of TIC sample size for the estimation.

### Genetic diversity and population structure

According to Decker et al. [29], Angus (AN) and Hereford (HFD), which are distributed across Europe, are considered as Eurasian *Bos taurus* populations; and GIR (GIR) and Sahiwal (SAHW) cattle, which are distributed across Asia, are considered as Asian *Bos indicus* populations. These breeds were selected because they are representative of commercial transboundary cattle and could be useful in this study to determine the extent of dilution from crossbreeding in Chinese cattle.

**Fig. 2:**
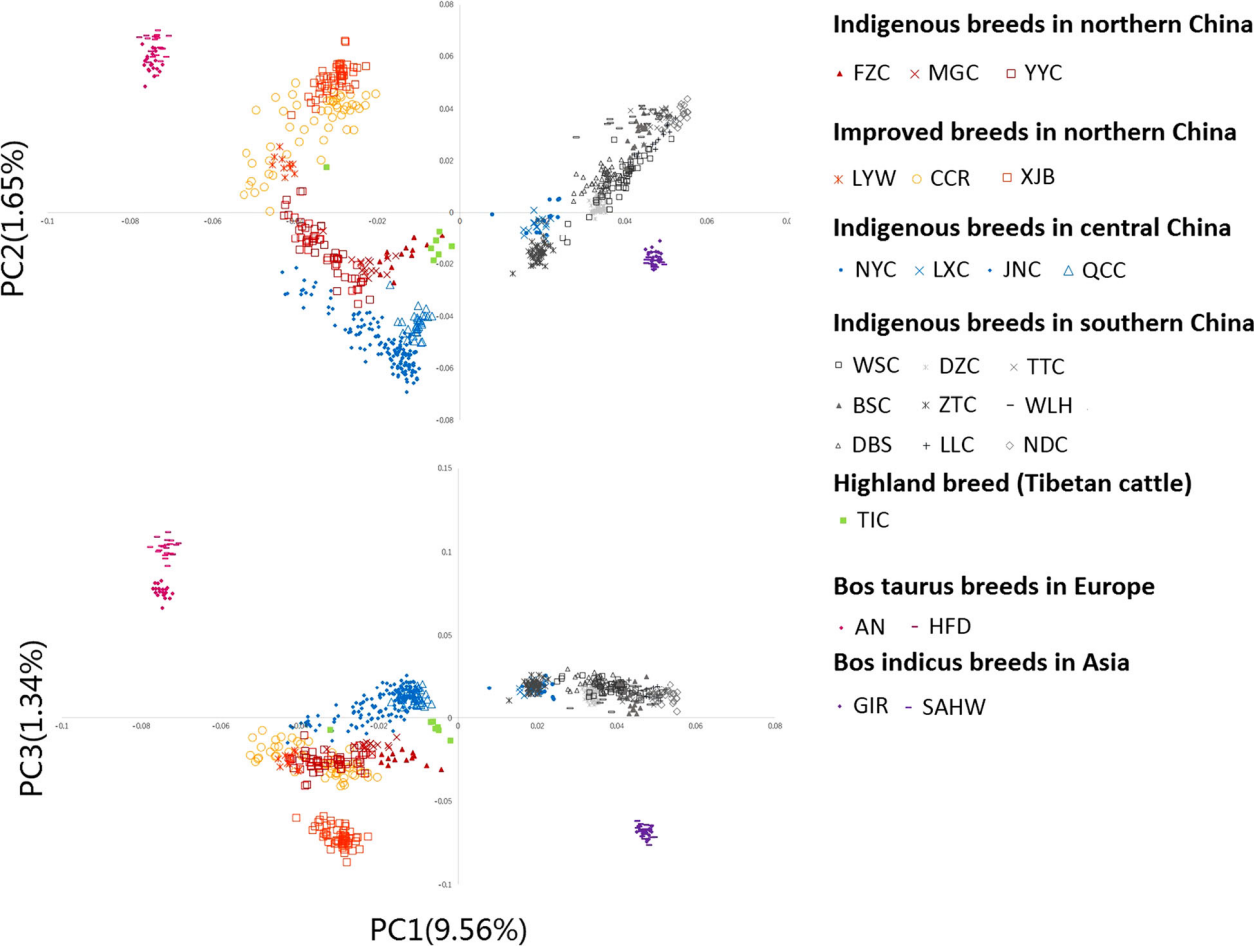
Principal component analysis of 630 individuals based on 7003 independent SNPs. PC1 explained 9.56% of global variation, PC2 explained 1.65% of global variation, and PC3 explained 1.34% of global variation. Light-red plots represent improved breeds in northern China, deep-red plots represent indigenous breeds in northern China, blue plots represent breeds in central China and grey plots represent breeds in southern China. For worldwide breeds, *Bos taurus* breeds and *Bos indicus* breeds are represented by purple-red plots and purple plots, respectively

Principal component analysis (PCA) was first conducted using 7003 merged SNPs in all 24 breeds. As shown in Fig. 2, the first principal component (PC1) explained 9.56% of the observed global variation and divided the samples into two clusters. Southern breeds (PC1 = 0.037 ± 0.010) were positioned to GIR/SAHW individuals (0.047 ± 0.011), and northern breeds (− 0.032 ± 0.009) were close to AN/HFD individuals (− 0.074 ± 0.0016). For breeds located in the central China, the individuals exhibited between southern and northern lineages, with NYC and LXC closer to southern breeds, on the contrary QCC and JNC closer to northern breeds. The second principal component (PC2), which accounted for 1.65% of global variation, separated some breeds from one another in the same regions (I YYC and CCR, II DZC and WSC). Notably, the third principal component (PC3), which explained 1.34% of the variation, clearly distinguished AN/HFD from northern populations as well as GIR/SAHW and southern populations. Overall, Chinese indigenous cattle were clustered into Eurasian taurine and Asian indicine lineages, consistent with geographic dispersal. For the worldwide breeds, AN/HFD (the upper left cluster in Fig. 2) had larger genetic differences to Chinese Northern breeds, compared with the genetic differences among those Northern breeds. Similar situation occurred in GIR and SAHW (the lower cluster in Fig. 2). Additionally, we conducted PCA analysis on all 20 Chinese breeds using 17,821 SNPs. The 18 K-PCA results had no significant difference to the 7 K-PCA results with R^2^_PC1_ > 0.95 and R^2^_PC2_ ≈ 0.9 (Additional file 1: Figure S3).

**Fig. 3:**
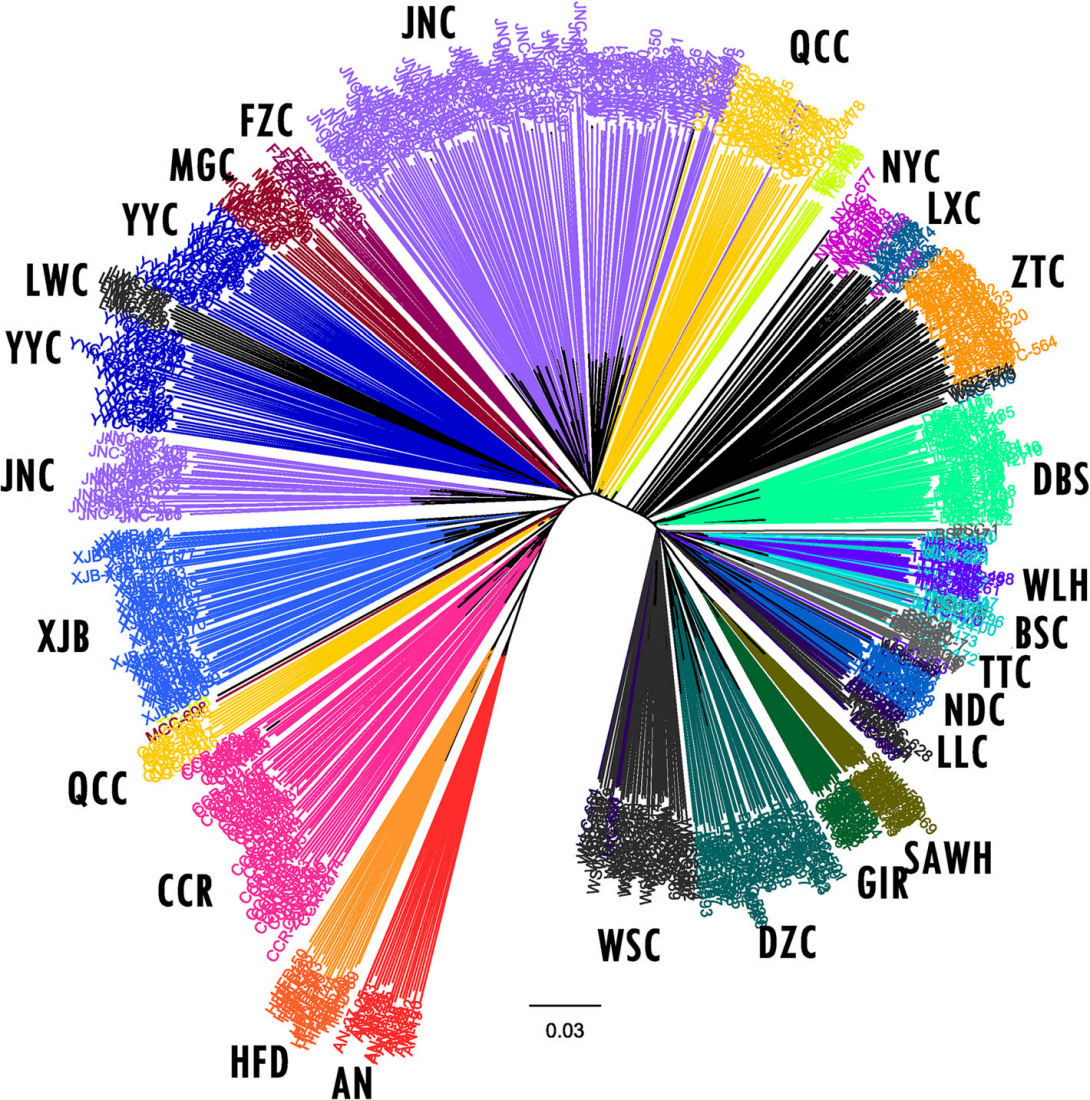
Neighbour-joining tree relating to the 630 individuals from twenty breeds of Chinese cattle. The tree was constructed using the allele sharing distance averaged over 7003 SNPs. Edges are coloured according to the individual breed of origin

We next constructed a neighbour-joining (NJ) tree using 7003 SNPs in all samples (Fig. 3). Individuals in the same breeds were roughly clustered together, especially the northern and southern species clearly separated. Individuals of QCC and JNC were divided into two distinct groups: a small part of samples mixed between European cattle (AN/HFD) and northern cattle, and the other samples situated in an intermediary position between northern and southern breeds. This division might be caused by uneven sample size, beadchip design or systematic error in the NJ tree. Another plausible explanation was that in recent years, QCC and JNC have crossbred with European cattle (such as Simmental), leading to the shared genetic backgrounds. For GIR and SAHW, they were located in a clade with Chinese southern cattle, which indicated that these breeds shared common ancestry. For AN and HFD, the two breeds were in the same clade with northern breeds but were relatively far from northern breeds compared with the distance among northern breeds. These results were consistent with the PC1 dimension results.

**Fig. 4:**
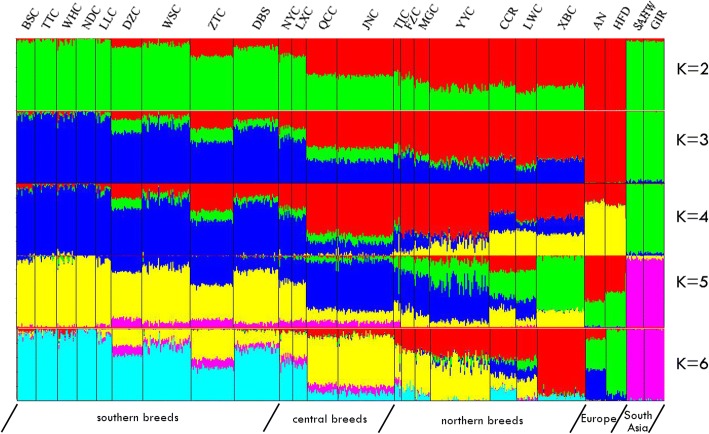
Model-based population assignment for 630 individuals based on 7003 SNPs using STRUCTURE (K = 2–6) and plotted with Distruct software. Eurasian *Bos taurus* represents worldwide breeds with European taurine ancestry, and *Bos indicus* represents worldwide breeds with zebu ancestry. For K = 2, red descent represents Asian indicine (*Bos indicus*) ancestry, and yellow descent represents European taurine (*Bos taurus*) ancestry

**Fig. 5:**
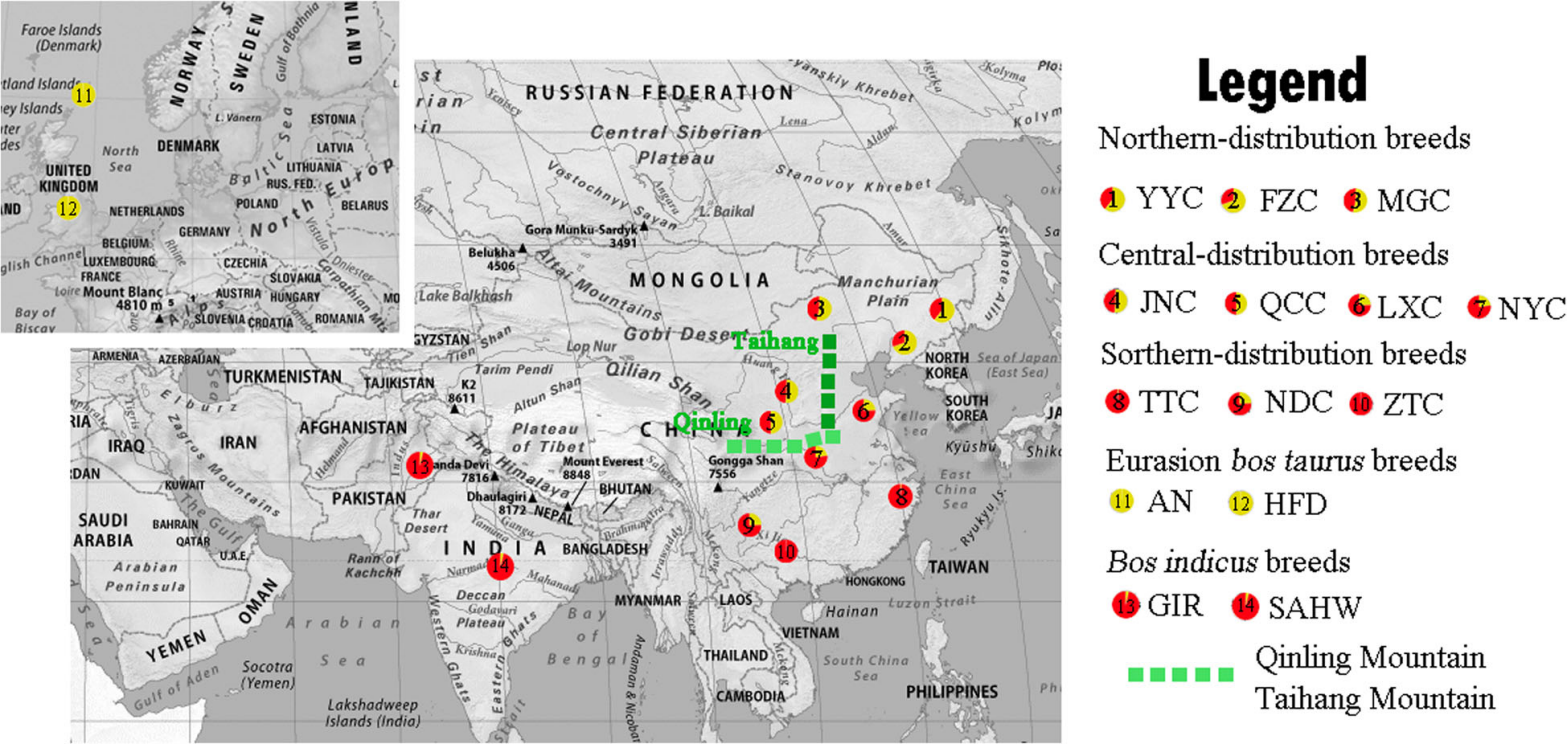
Localities of cattle breeds and the frequency of Eurasian taurine and Asian indicine lineages. The Qinling Mountains and Taihang Mountains are represented with green lines. (Map is downloaded from https://www.mapsofworld.com/asia/)

To provide additional insight into the genetic variation and admixture of Chinese indigenous cattle, we used STRUCTURE software to conduct model-based clustering of all individuals [31]. At K = 2 (Fig. 4), we found that the worldwide breeds GIR and SAHW exhibited an average of 96.5% taurine content, whereas for breeds AN and HFD, the average of 99.1% taurine content was shown. In Chinese breeds, southern indigenous populations (BSC, TTC, WHC, NDC, LLC, DZC, WSC, ZTC, and DBS) displayed a high level of indicine ancestry, instead northern populations (FZC, MGC, YYC, CCR, LWC and XJB) and TIC tend to more influenced by taurine. In Fig. 5, the distribution of population and frequency of ancestry were shown when K = 2 in some indigenous breeds. At K = 3, we found a distinct descent within indicine that influenced most individuals from southern breeds. Notably, six southern breeds (BSC, TTC, WHC, NDC, LLC, and WSC) contained more than 90% of this genetic descent, and northern breeds were also affected by this descent, with 31.56% (on average) of total genomes. At K = 4, 5 and 6, Chinese breeds were admixed with several assumed lineages in a complicated fashion, and no breed separated from the others independently, which is reasonable, because unlike worldwide commercial breeds that have experienced mild artificial selection for a long time, Chinese indigenous breeds continuously experience neutral selection in their surroundings without extreme environmental selection or artificial selection. We also found that the influence of indicine remained in major breeds in southern and central areas with 8–15% of total genomes. For the suitable K value, Structure Harvester analysis suggested K = 5 as the most likely number of genetically distinct groups within our samples (Additional file 1: Figure S4).

### Admixture analysis

**Fig. 6:**
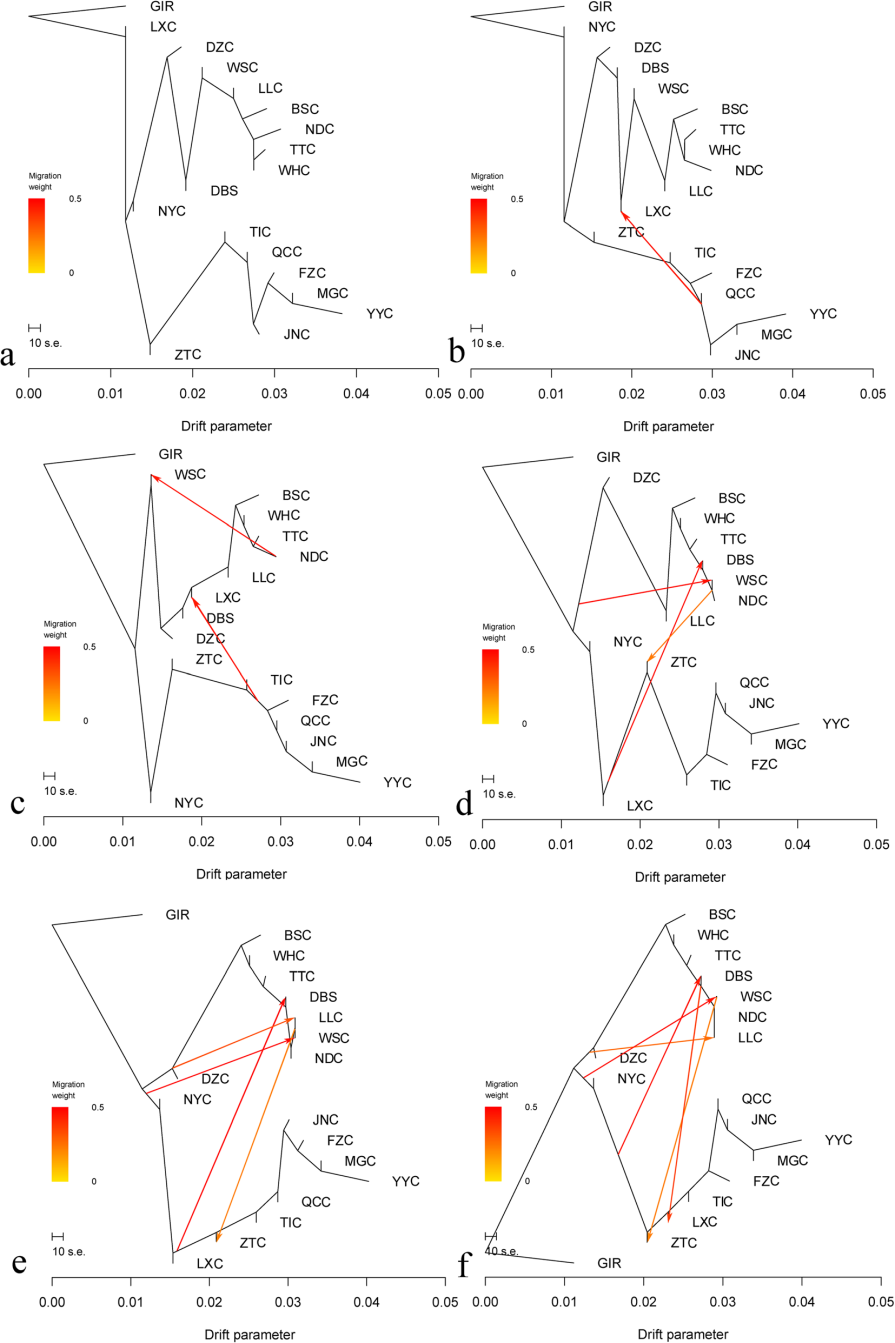
Maximum likelihood tree inferred from 18 cattle populations with migration events**.** a, no migration events; b-f, one to five migration events, respectively. Migration arrows are coloured according to their weight

**Fig. 7:**
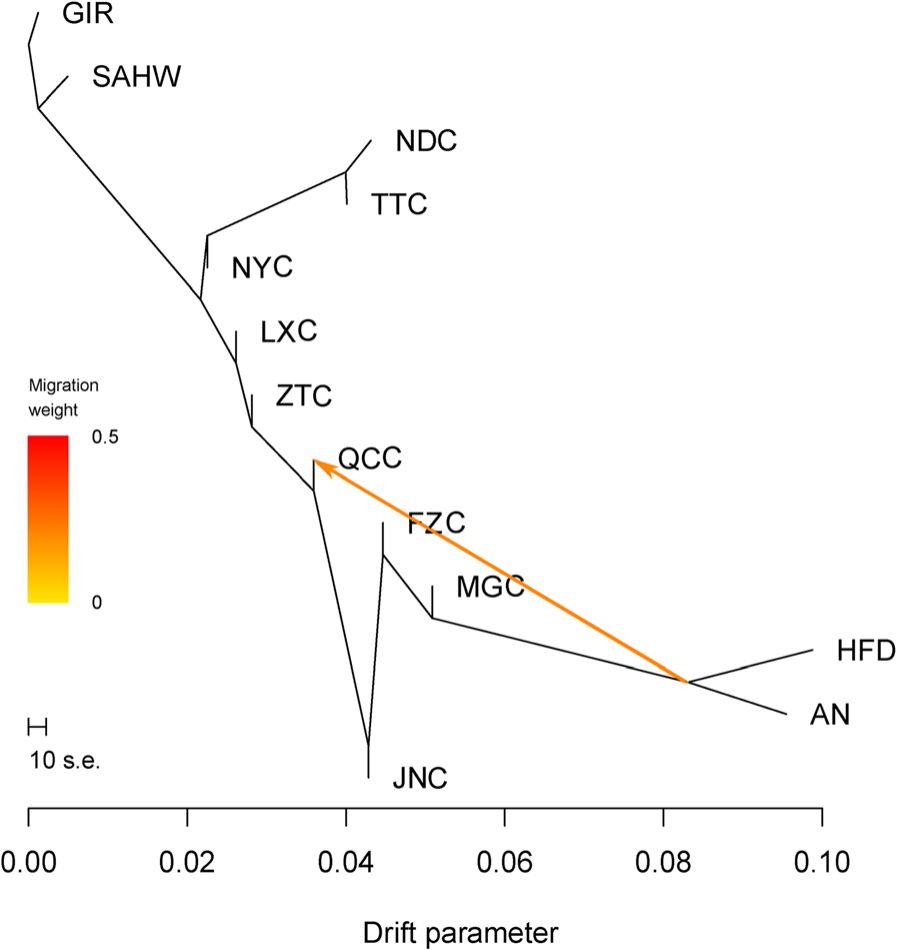
Maximum likelihood tree inferred from 13 cattle breeds with one migration event

To explore the patterns of divergence of indigenous cattle, we used TreeMix software to model both population splits and gene flow among a subset of 18 populations (17 indigenous Chinese cattle and GIR as the root). A phylogenetic tree without migration events was constructed based on 7003 SNPs (Fig. 6-a). All 17 indigenous populations were clustered into two primary branches representing a northern lineage and a southern lineage, with breeds from the central region splitting between these two clusters. However, the central-distributed breeds, LXC and NYC, were sister groups to southern breeds ancestry, which is a contradicted result with PCA and STRUCTURE. Therefore, when one and two migration events were added, we observed an influence of northern lineage on LXC (Fig. 6-b, c), indicating introgression events occurred from northern breeds to LXC historically. With the number of migration event increasing, gene flows were detected from southern or northern lineage to BSC, WSC, ZTC, and LLC (Fig. 6-d, e, f, g), suggesting that the messy introgression events occurred in southern breeds. As we known, to enhance the production capability of offspring, QCC and JNC were improved by introduction of European commercial breeds blood  through artifical insemination in 1970s. Notably, this migration event was clearly detected using TreeMix analysis (Fig. 7) based on thirteen breeds (9 Chinese indigenous breeds, AN, HFD, GIR, and SAHW).

To confirm the admixture of the central populations with the northern and southern populations, we applied the 3 Population Test (*f3*) using TreeMix software (Table 2). Populations in central areas (QCC, JNC, LXC, and NYC) were analysed since the greater possibility of admixture events occurred among northern and southern populations resulting in central populations. Except for JNC, the *f3* statistics were all negative, which suggested the admixture of southern and northern breeds. All above of these results were consistent with those of STRUCTURE.

**Table 2:** Results of the *f3* for Chinese indigenous cattle^1^

X population	Y population	Z population	*f3* statistics	Z-score from *f3*
QCC^2^	NDC	MGC	− 0.0017	−4.8663
QCC	TTC	MGC	−0.0014	−4.33679
NYC	NDC	MGC	−0.0064	−18.3264
NYC	TTC	MGC	−0.0047	−12.8435
LXC	TTC	FZC	−0.0053	−13.0848
LXC	TTC	MGC	−0.0080	−32.1615
LXC	TTC	JNC	−0.0084	−56.7148
LXC	TTC	QCC	−0.0082	−62.5641
JNC	TTC	MGC	0.0005	1.6375
JNC	NDC	MGC	0.0014	6.2444

^1^Three populations are included in the topology structure. If the f3 statistics result is significant negative, the X population may have descended from an admixture event of the Y and Z populations

^2^See Table 1 for breed abbreviations

## Discussion

### Population structure in Chinese breeds

Previous studies indicate that Eurasian taurine cattle spread into northern China between approximately 3000 and 2000 BC and appeared in the central plains between 2500 and 1900 BC; by contrast, indicine cattle inhabited the south of China no earlier than 1500 BC [16–19]. Here, based on PCA and NJ tree analysis, our results are consistent with previous studies that northern breeds are clustered together and have close relation to AN and HFD, indicating an origin of taurine lineage. Additionally, southern individuals covered GIR and SAHW in the PC1 dimension and shared the clade with GIR and SAHW in the NJ tree, indicating an origin of indicine lineage. Consistent with the results of PCA and NJ tree, STRUCTURE (K = 2) suggested that migration events of *Bos indicus* and Eurasian *Bos taurus* historically shaped southern and northern indigenous breeds, respectively. However, at K = 3, a distinct descent replaced indicine descent in most southern breeds, which indicated that an initial admixture event occurred between Chinese cattle and widely dispersed indicine breeds. As one plausible explanation for the huge influence of this descent, Chinese indigenous cattle underwent different selection pressures for thousands of years that caused differentiation between Chinese and indicine cattle. Notably, when K = 4, 5, and 6, we detected a stable influence of indicine on individuals of DZC (averaged *15.0%*, SD *1.9%*), WSC (*8.7*, *2.2%*), ZTC (*13.9*, *1.4%*), DBS (*7.9*, *1.5%*), NYC (*11.1*, *2.0%*), LXC (*11.8*, *1.9%*), QCC (*11.9*, *1.6%*) and JNC (*10.7*, *2.1%*). According to these results, we inferred a second migration event that might have occurred in modern time from Zebu breeds to these breeds.

Northern Chinese indigenous breeds, which are widely distributed in the north and northeast of China, are generally thought to have originated from *Bos taurus* and migrated from East Asia [15, 17, 32, 33]. Based on microsatellite markers of the Chinese indigenous populations [12, 15, 34], these researches led to the conclusion that northern indigenous breeds had more than 90% Eurasian *Bos taurus* descent. However, in our study, based on worldwide and indigenous cattle breeds genotyped with markers, the northern indigenous breeds were estimated to exhibit only 62.70% (±8.33) *Bos taurus* lineage introgression on average. Therefore, we speculated that a greater *Bos indicus* introgression occurred in Chinese northern indigenous cattle than that reported in previous studies.

### Admixture in central breeds and natural barriers

To reflect the genetic diversity and the pattern of admixture, *P*n, *MAF*, and *H*o were tested in Chinese indigenous breeds. Because these analyses were unavoidably heavily reliant on sample characteristics (sample size or relationships among individuals) and selected SNPs, we used a bootstrapping method. Compared with northern-distributed and southern-distributed breeds, central-distributed breeds were more polymorphic with the highest *P*n, *MAF*, and *H*o, indicating these breeds might be the admixture of southern and northern cattle, or other genetic materials might have been introduced into central breeds in recent time. According to STRUCTURE (K = 2 and 3) and PCA results, the assumption that central breeds were shaped by admixture was supported. For further exploration, we conducted *f3* statistics to validate the hypothesis that central area breeds descended from mixtures of northern and southern populations. Only one central area breed, JNC, did not present a significant signal of mixture according to the *f3* analysis. The lack of a significant result did not demonstrate that JNC was not mixed but indicated that there was a substantial genetic drift occurred in JNC, which was supported by the observation that JNC presented a minimum *F*_*st*_ of 0.025 among all other indigenous breeds (Additional file 1: Table S1).

According to the results analyzed by mtDNA and Y-chromosome, Cai et al. have proved that the genetic divergence between southern and northern breeds can be attributed to geographical segregation of Qinling Mountains. In this study, based on the STRUCTURE (K = 2) results, QCC and JNC (located in the north of the Qinling Mountains) displayed 31.8 and 30.8% indicine contents, respectively, whereas NYC (located in the south of the Qinling Mountains) presented a 62.4% indicine content. The lineage proportions of these three breeds are consistent with the hypothesis. Furthermore, LXC (distributed to the east of the Taihang Mountains) showed a 60.0% indicine content, suggesting that the Taihang Mountains may constitute natural barriers to cattle’s expansion in China, which needs further investigation. Notably, although northern breeds were hampered in the flow to the south direction by natural barriers, we observed several migration events from northern breeds to LXC in TreeMix (Fig. 6). We inferred that these results indicate natural migration and artificial hybridization with European breeds such as in QCC and JNC.

### Tibetan cattle admixture lineage

After quality control, thirteen TIC individuals were removed due to call rate less than 0.95, of which ten samples were less than 0.8. This result indicated that most of the TIC samples we collected might be from *dzo*, a hybrid of the yak and Tibetan cattle [35].

As one of the distinct indigenous breeds, Tibetan cattle are distributed in southern and eastern Tibet at an altitude of over 4000 m [36]. Lei et al. [37] and Zhang et al. [12] suggested that Tibetan cattle originated from Eurasian *Bos taurus* and Asian *Bos indicus* and could be classified into the northern group of Chinese cattle. In our study, TIC individuals were clearly separated from other breeds in both the PCA (Fig. 2) and NJ tree analysis (Fig. 3), and STRUCTURE result (Fig. 4) showed admixed ancestry.

## Conclusions

In this study, Chinese indigenous cattle were divided into two genetic clusters, corresponding to northern indigenous cattle (Eurasian taurine lineage) and southern indigenous cattle (Asian indicine lineage), which are consistent with the observed geographic distributions. Three improved breeds in the northern area also exhibited the taurine lineage. The results of TreeMix and the *f3* analysis revealed a history of admixture of central breeds and suggested introgression events in Chinese indigenous cattle. Our study provides a comprehensive overview of the population structure and genetic diversity of Chinese indigenous cattle breeds, and the results help to further investigate the genetic resources underlying adaptation traits in these breeds.

## Methods

### Cattle populations, DNA samples and SNP genotyping

We collected 572 blood samples from twenty Chinese indigenous cattle breeds following approval by the Agriculture and Animal Husbandry Bureaus of local areas. In this study, all domestic cattle owned by the local institute or farmers has been authorized by the local livestock department of government for scientific research. Table 1 showed the full names and abbreviations, sample sizes, and geographic regions for each breed. DNA was extracted using a TIANamp Blood DNA Kit (Tiangen Biotech Company Ltd., Beijing, China), and qualified DNA samples were genotyped using GeneSeek Genomic Profiler Bovine LD (GGP-LD) assays (N_SNP_ = 30,125). Additionally, published data from 4 commercial breeds genotyped with the Illumina BovineSNP50 Beadchip [29] were downloaded (10.5061/dryad.th092) including Angus (20), Hereford (20), GIR (20), and Sahiwal (17).

### Quality control and genetic diversity analyses

Quality control was performed using PLINK 1.7 software [38] to remove SNPs showing a call rate of less than 95%, a minor allele frequency (MAF) of less than 0.01 or significant deviation from Hardy-Weinberg equilibrium (*P* < 10^− 5^). Moreover, samples with more than 10% missing genotypes were removed from the data set. We also excluded related SNPs using the --indep-pairwise option, with a window size of 50 SNPs, a step of 10 SNPs, and an r^2^ threshold of 0.1. Finally, we obtained 7003 independent SNPs for following analysis.

The proportion of polymorphic SNPs (*P*n) indicates the fraction of total SNPs that displays both alleles within each population. Depending on 7003 SNPs, the expected heterozygosity (*H*e), observed heterozygosity (*H*o), and inbreeding coefficient (*F*) were estimated with PLINK. ANOVAs were implemented to compare the differences among cattle breeds with SPSS.

The recent effective population size (*N*e) for each breed was estimated using software NeESTIMATOR v2 [39]. The final *N*e estimates were bias-corrected values using LD method [40].

### Population analyses

PCA was implemented using the R 3.2.1 program (https://cran.r-project.org/bin/windows/base/) with 7003 independent SNPs in all 24 breeds. The genetic distance matrix between pair-wise individuals was calculated using the PLINK --distance-matrix option, and an individual NJ tree was constructed using PHYLIP [41] software. Population structure was analysed for K = 2–6 using STRUCTURE 2.3.4 software [31] and plotted using Distruct software [42]. All analyses were performed with a burn-in length of 30,000, followed by 50,000 MCMC replications for each K value. The TreeMix software package [43] was employed for phylogenetic analyses to investigate interpopulation migration and gene flow, and *f3* statistics were tested with the TreeMix software using 7003 independent SNPs. The *F*_*st*_ values among breeds were calculated based on the unbiased estimator form illustrated by Weir and Cockerham [44] using Genepop software.

## Additional file

Additional file 1: **Figure S1.** Comparison of proportion of polymorphic (*Pn*). **Figure S2.** Comparison of minor allele frequency (MAF) and boxplot of MAF in each breed. **Figure S3.** Comparison between 18 K-dataset and 7 K-dataset in Principal component analysis. **Figure S4.** Delta K values for STRUCTURE analysis of Chinese indigenous cattle using Evanno method. **Table S1.** Autosomal average Fst among 20 Chinese cattle breeds. (DOCX 477 kb)

## Abbreviations

DNA: Deoxyribonucleic acid
F: Inbreeding coefficient
He: Expected heterozygosity
Ho: Observed heterozygosity
LD: Linkage disequilibrium
NJ: Neighbour-Joining
PCA: Principal component analysis
Pn: Proportion of polymorphic SNPs
SNP: Single nucleotide polymorphism
