# Supplementary material for: Genome-wide assessment of genetic diversity and population structure insights into admixture and introgression in Chinese indigenous cattle
Source: BMC Genet. 2018 Dec 20;19:114. doi: 10.1186/s12863-018-0705-9 (PMC6302425; doi:10.1186/s12863-018-0705-9)
Supplement: Supplementary file 1 — Figure S1. Comparison of proportion of polymorphic (Pn). Figure S2. Comparison of minor allele frequency (MAF) and boxplot of MAF in each breed. Figure S3. Comparison between 18 K-dataset and 7 K-dataset in Principal component analysis. Figure S4. Delta K values for STRUCTURE analysis of Chinese indigenous cattle using Evanno method. Table S1. Autosomal average Fst among 20 Chinese cattle breeds. (DOCX 477 kb) [file 12863_2018_705_MOESM1_ESM.docx]

**Genome-wide assessment of genetic diversity and population structure insights into admixture and introgression in Chinese indigenous cattle**

Wengang Zhang^1*^, Xue Gao^1*^, Yang Zhang^2^, Yumin Zhao^3^, Jiabao Zhang^4^, Yutang Jia^5^, Bo Zhu^1^, Lingyang Xu^1^, Lupei Zhang^1^, Huijiang Gao^1^, Junya Li^1**^, Yan Chen^1**^

^1^Innovation Team of Cattle Genetics and Breeding, Institute of Animal Science (IAS), Chinese Academy of Agricultural Sciences (CAAS), Beijing 100193, China.

^2^Xinjiang Academy of Animal Science, Urumqi 830011, China.

^3^Jilin Academy of Animal Science, Changchun 130124, China.

^4^Jilin University, Changchun 130012, China.

^5^Institute of Animal Husbandry and Veterinary Medicine, AnhuiAcademyof Agricultural Sciences, Hefei 230031, China.

^*^These authors contributed equally to this work.

****Corresponding authors:**

**Yan Chen**

Address: Institute of Animal Science, Chinese Academic of Agriculture Science, Yuanmingyuan West Road 2#, Haidian District, Beijing, China.

Telephone: +86-134-3967-4745

Email: [chenyan0204@163.com](mailto:chenyan0204@163.com)

**Junya Li**

Address: Institute of Animal Science, Chinese Academic of Agriculture Science, Yuanmingyuan West Road 2#, Haidian District, Beijing, China.

Telephone: +86-138-1156-8766

Email: [lijunya@caas.cn](mailto:lijunya@caas.cn)

**Running Title**: Genome-wide analysis revealed diversity, structure, and introgression of indigenous cattle in China

**Counts**: 6617 words Reference: 44 Tables: 2 Figures: 7

Additional file 1

Figure S1. Comparison of proportion of polymorphic (Pn).

Figure S2. Comparison of minor allele frequency (MAF) and boxplot of MAF in each breed.

Figure S3. Comparison between 18K-dataset and 7K-dataset in Principal component analysis.

Figure S4. Delta K values for STRUCTURE analysis of Chinese indigenous cattle using Evanno method.

Table S1. Autosomal average Fst among 20 Chinese cattle breeds.


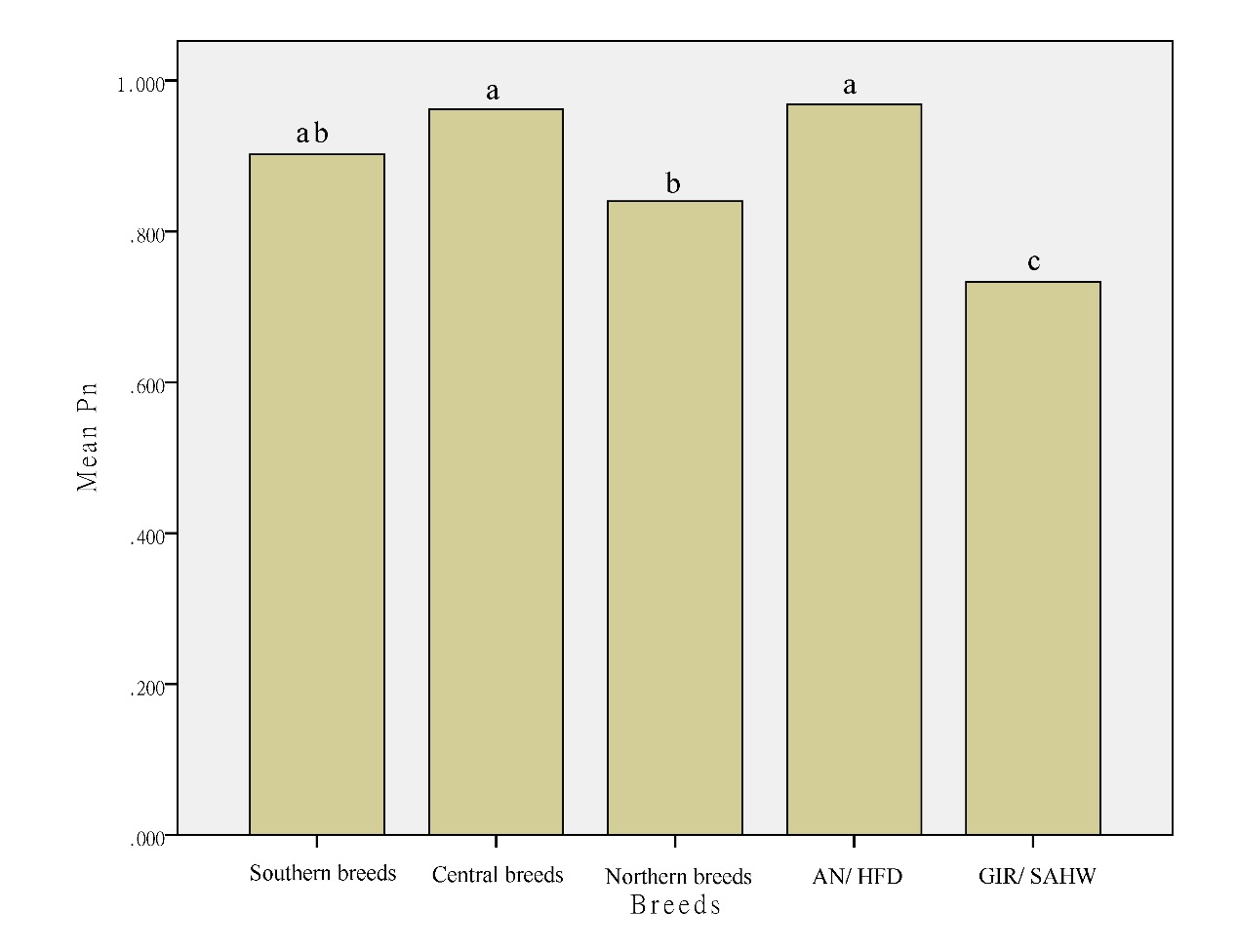


**Figure S1. Comparison of proportion of polymorphic (*Pn*).**


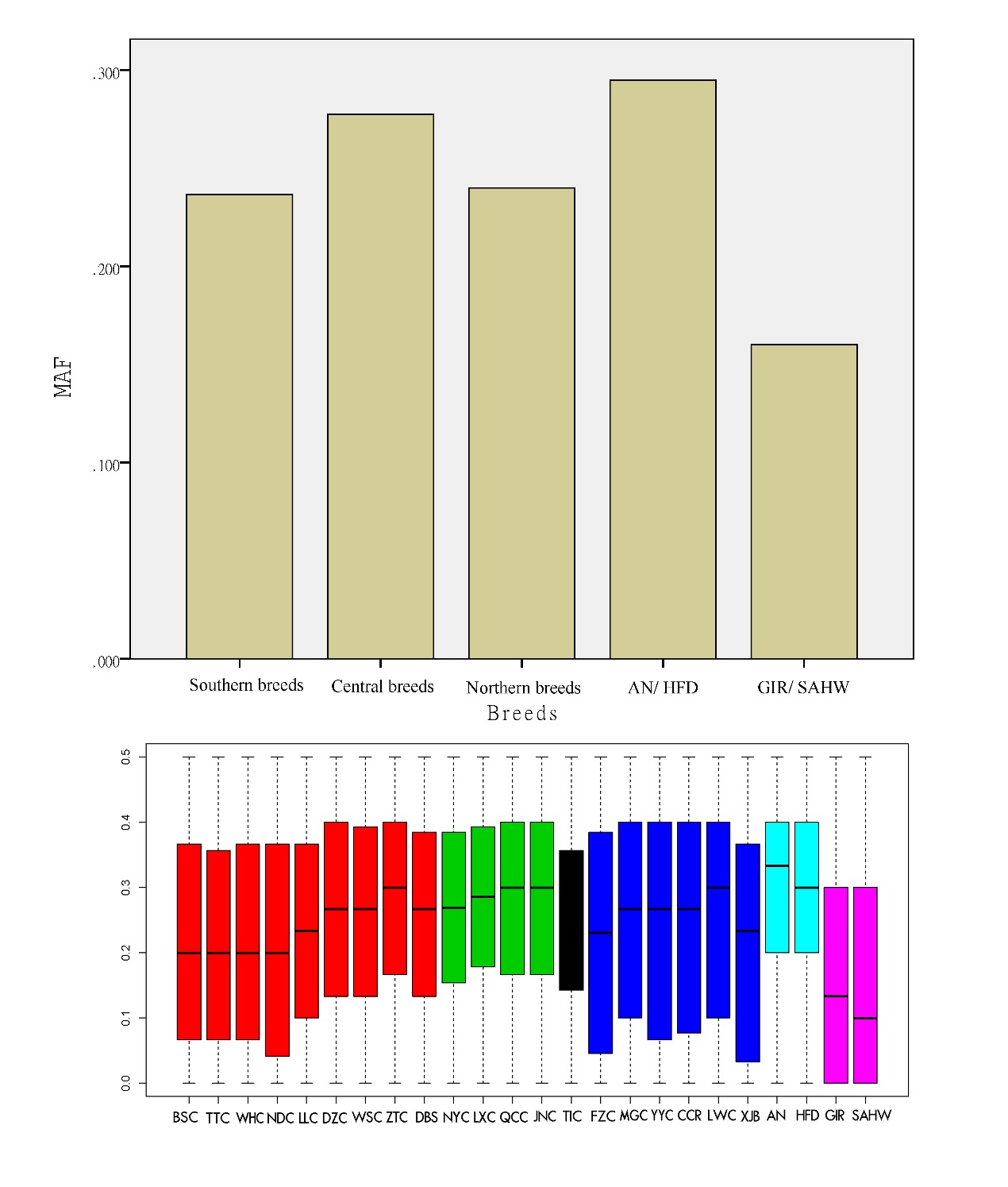


**Figure S2. Comparison of minor allele frequency (MAF) and boxplot of MAF in each breed.** Southern breeds in red, central breeds in green, northern breeds in blue, Angus and Hereford in light blue, and GIR and Sahiwal in pink.

**
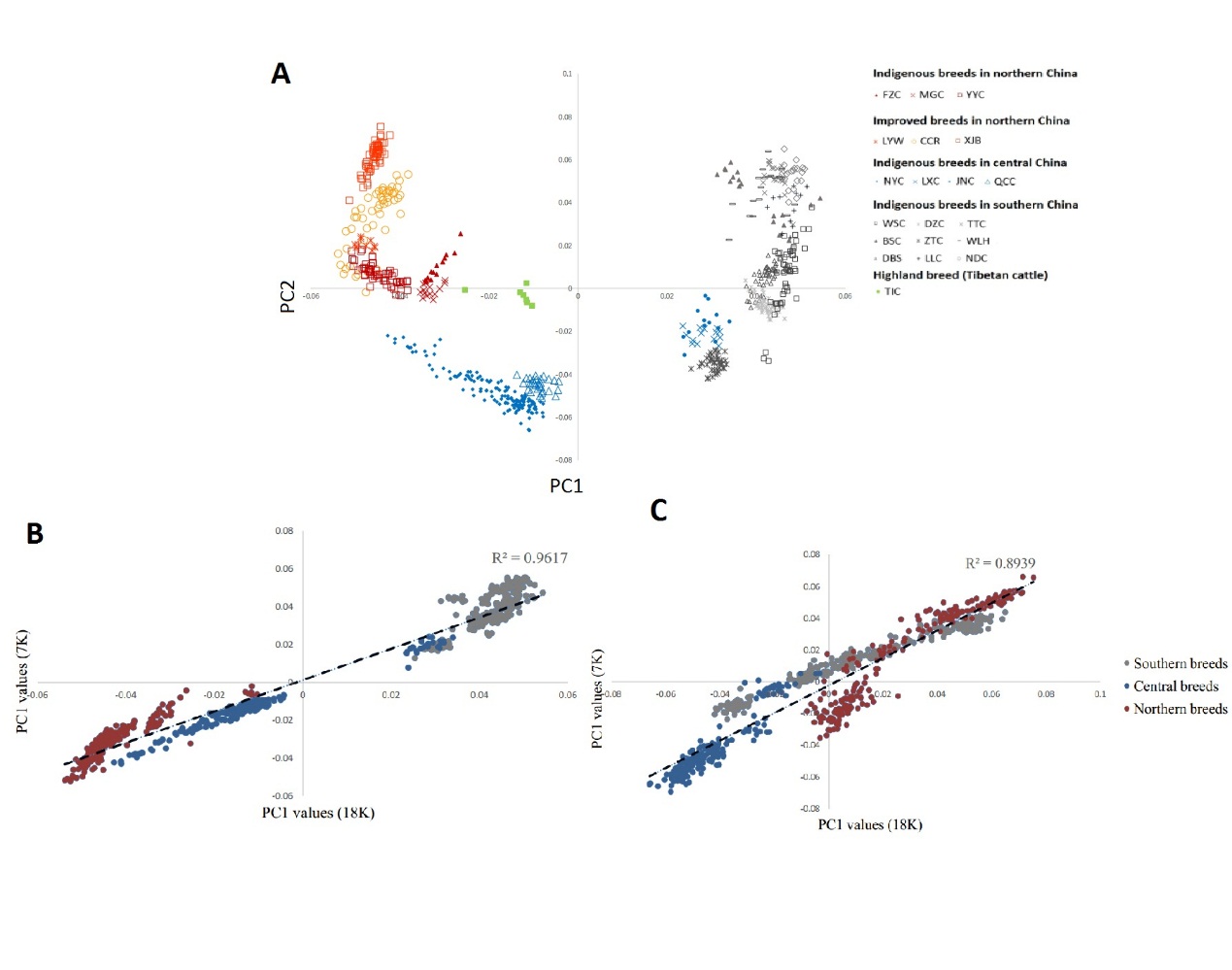
**

**Figure S3. Comparison between 18K-dataset and 7K-dataset in Principal component analysis.**

A) Principal component analysis of Chinese indigenous breeds genotyped with 17,821 SNPs.

B) Comparison of PC1 value between 18K dataset and 7K dataset (red, blue and grey plots represent individuals belonging to northern-distributed breeds, central-distributed breeds and southern-distributed breeds, respectively).

C) Comparison of PC2 value between 18K dataset and 7K dataset.


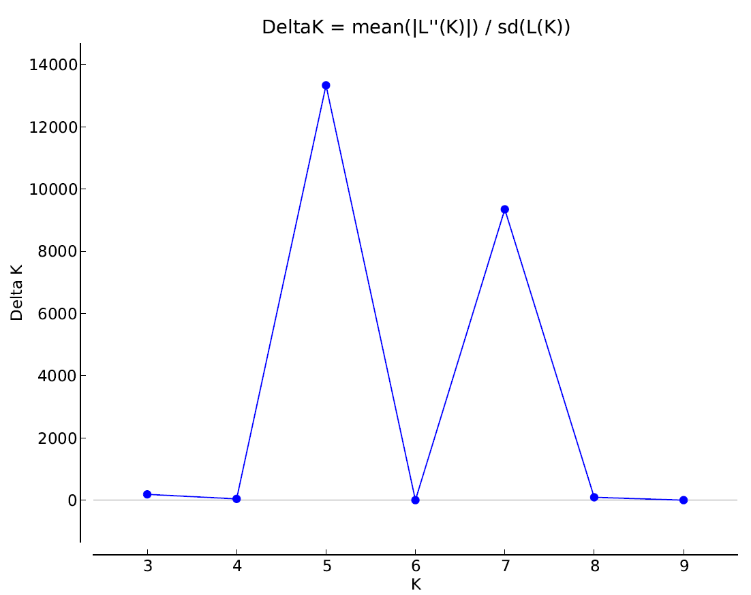


**Figure S4. Delta K values for STRUCTURE analysis of Chinese indigenous cattle using Evanno method.**

Table S1. Autosomal average Fst among 20 Chinese cattle breeds.

|  | TTC | WHC | NDC | LLC | DZC | WSC | ZTC | DBS | NYC | LXC | QCC | JNC | TIC | FZC | MGC | YYC | CCR | LWC | XJB |
| --- | --- | --- | --- | --- | --- | --- | --- | --- | --- | --- | --- | --- | --- | --- | --- | --- | --- | --- | --- |
| BSC | 0.025 | 0.022 | 0.028 | 0.029 | 0.024 | 0.022 | 0.028 | 0.021 | 0.045 | 0.039 | 0.050 | 0.029 | 0.055 | 0.075 | 0.072 | 0.058 | 0.062 | 0.080 | 0.072 |
| TTC |  | 0.012 | 0.020 | 0.023 | 0.023 | 0.020 | 0.029 | 0.019 | 0.043 | 0.038 | 0.052 | 0.032 | 0.053 | 0.072 | 0.071 | 0.061 | 0.065 | 0.081 | 0.073 |
| WHC |  |  | 0.022 | 0.023 | 0.021 | 0.018 | 0.025 | 0.017 | 0.040 | 0.033 | 0.046 | 0.027 | 0.049 | 0.070 | 0.066 | 0.058 | 0.061 | 0.075 | 0.071 |
| NDC |  |  |  | 0.018 | 0.021 | 0.017 | 0.028 | 0.018 | 0.045 | 0.039 | 0.055 | 0.032 | 0.058 | 0.077 | 0.076 | 0.061 | 0.065 | 0.084 | 0.075 |
| LLC |  |  |  |  | 0.014 | 0.012 | 0.018 | 0.013 | 0.042 | 0.031 | 0.041 | 0.022 | 0.058 | 0.081 | 0.073 | 0.053 | 0.058 | 0.076 | 0.070 |
| DZC |  |  |  |  |  | 0.012 | 0.014 | 0.015 | 0.018 | 0.016 | 0.029 | 0.029 | 0.021 | 0.039 | 0.036 | 0.061 | 0.064 | 0.053 | 0.073 |
| WSC |  |  |  |  |  |  | 0.012 | 0.009 | 0.018 | 0.014 | 0.030 | 0.026 | 0.024 | 0.045 | 0.040 | 0.062 | 0.065 | 0.057 | 0.076 |
| ZTC |  |  |  |  |  |  |  | 0.014 | 0.015 | 0.011 | 0.018 | 0.018 | 0.017 | 0.035 | 0.028 | 0.051 | 0.055 | 0.043 | 0.067 |
| DBS |  |  |  |  |  |  |  |  | 0.016 | 0.012 | 0.028 | 0.024 | 0.024 | 0.044 | 0.038 | 0.059 | 0.062 | 0.055 | 0.072 |
| NYC |  |  |  |  |  |  |  |  |  | 0.028 | 0.029 | 0.014 | 0.059 | 0.081 | 0.065 | 0.043 | 0.049 | 0.065 | 0.064 |
| LXC |  |  |  |  |  |  |  |  |  |  | 0.021 | 0.013 | 0.037 | 0.063 | 0.048 | 0.047 | 0.052 | 0.057 | 0.067 |
| QCC |  |  |  |  |  |  |  |  |  |  |  | 0.006 | 0.020 | 0.035 | 0.023 | 0.026 | 0.032 | 0.031 | 0.045 |
| JNC |  |  |  |  |  |  |  |  |  |  |  |  | 0.009 | 0.016 | 0.011 | 0.022 | 0.028 | 0.018 | 0.037 |
| TIC |  |  |  |  |  |  |  |  |  |  |  |  |  | 0.044 | 0.034 | 0.014 | 0.018 | 0.029 | 0.026 |
| FZC |  |  |  |  |  |  |  |  |  |  |  |  |  |  | 0.037 | 0.014 | 0.020 | 0.035 | 0.024 |
| MGC |  |  |  |  |  |  |  |  |  |  |  |  |  |  |  | 0.011 | 0.016 | 0.025 | 0.025 |
| YYC |  |  |  |  |  |  |  |  |  |  |  |  |  |  |  |  | 0.014 | 0.011 | 0.021 |
| CCR |  |  |  |  |  |  |  |  |  |  |  |  |  |  |  |  |  | 0.013 | 0.021 |
| LWC |  |  |  |  |  |  |  |  |  |  |  |  |  |  |  |  |  |  | 0.021 |
